# Main Cardiac Histopathologic Alterations in the Acute Phase of *Trypanosoma cruzi* Infection in a Murine Model

**DOI:** 10.3390/pathogens12091084

**Published:** 2023-08-26

**Authors:** Mariana C. de Alba Alvarado, Elia Torres Gutiérrez, Margarita Cabrera Bravo, Edgar Zenteno Galindo, José Antonio Villarreal Muñoz, Paz María Salazar Schettino, Martha Irene Bucio Torres

**Affiliations:** 1 Departamento de Microbiología y Parasitología, Facultad de Medicina, Universidad Nacional Autónoma de México, Coyoacán, Mexico City 04510, Mexico; marianadealba@comunidad.unam.mx (M.C.d.A.A.); etorres@facmed.unam.mx (E.T.G.); imay@unam.mx (M.C.B.); 2 Departamento de Bioquímica, Facultad de Medicina, Universidad Nacional Autónoma de México, Coyoacán, Mexico City 04510, Mexico; ezenteno@unam.mx; 3 División de Investigación, Secretaria General, Facultad de Medicina, Universidad Nacional Autónoma de México, Coyoacán, Mexico City 04510, Mexico; avillarreal@unam.mx

**Keywords:** acute chagas disease, cardiac histopathology, pathogenicity, mouse model

## Abstract

Symptoms in the acute phase of Chagas disease are usually mild and nonspecific. However, after several years, severe complications like dilated heart failure and even death may arise in the chronic phase. Due to the lack of specific symptoms in the acute phase, the aim of this work was to describe and analyze the cardiac histopathology during this phase in a CD1 mouse model by assessing parasitism, fibrotic damage, and the presence and composition of a cellular infiltrate, to determine its involvement in the pathogenesis of lesions in the cardiac tissue. Our results indicate that the acute phase lasts about 62 days post-infection (dpi). A significant increase in parasitemia was observed since 15 dpi, reaching a maximum at 33 dpi (4.1 × 10^6^). The presence of amastigote nests was observed at 15–62 dpi, with a maximum count of 27 nests at 35 dpi. An infiltrate consisting primarily of macrophages and neutrophils was found in the cardiac tissue within the first 30 days, but the abundance of lymphocytes showed an 8 ≥ fold increase at 40–62 dpi. Unifocal interstitial fibrosis was identified after 9 dpi, which subsequently showed a 16 ≥ fold increase at 40–60 dpi, along with a 50% mortality rate in the model under study. The increased area of fibrotic lesions revealed progression in the extent of fibrosis, mainly at 50–62 dpi. The presence of perivasculitis and thrombus circulation disorders was seen in the last days (62 dpi); finally, cases of myocytolysis were observed at 50 and 62 dpi. These histopathological alterations, combined with collagen deposition, seem to lead to the development of interstitial fibrosis and damage to the cardiac tissue during the acute phase of infection. This study provides a more complete understanding of the patterns of histopathological abnormalities involved in the acute phase, which could help the development of new therapies to aid the preclinical tests of drugs for their application in Chagas disease.

## 1. Introduction

Chagas disease is clinically characterized by an acute phase lasting 2–3 weeks, and a chronic phase with varying duration. The latter can be either asymptomatic or symptomatic, depending on the presence of demonstrated pathology. During the acute phase, signs and symptoms are related to the route of entry of the parasite, such as the Romaña sign or inoculation chagoma. The most affected organ in the chronic phase is the heart, where a cardiomyopathy dilated with cardiomegaly is the main presentation, usually manifested by progressive cardiac insufficiency and cardiac rhythm disorders [1,2]. In the acute phase, after the primary infection, myocardial involvement is described with intracellular replication of the parasite in myocytes, lysis, and the onset of an inflammatory process that progresses to the fibrosis which characterizes the chronic phase [3,4]. Various studies have directly related myocardial fibrosis to the inflammatory response, consisting primarily of lymphocytes and macrophages [5,6].

Several mammalian species were used to study the pathogenesis of cardiac lesions in Chagas disease; however, there are no descriptions of the presence of histopathological alterations after primary infection that would allow us to determine the onset of fibrosis generation during the acute phase and its progression to the chronic phase of the disease, for which it is distinctive. In this study, the histopathological characterization of cardiac lesions was performed in a murine model (CD1) during the acute phase, over 62 days after primary infection with *Trypanosoma cruzi*.

## 2. Materials and Methods

### 2.1. Parasites

A *T. cruzi* isolate from the state of Querétaro (ITRI/MX/1986/QRO), which is described as highly virulent in mice and probably related to human cases, was used herein. It is maintained at the Laboratory of Parasite Biology of the Faculty of Medicine, UNAM. This strain was isolated by our research group in 1986 from *Triatoma barberi* in an epidemiological study conducted in the state of Querétaro [7].

### 2.2. Infection in a Murine Model

The CD1 mouse strain (*Mus musculus*), a well-characterized outbred strain, was used. In total, 80 female mice, weighing 28–32 g (6.5 weeks old), were used; five animals were kept per cage. Each mouse was inoculated by intraperitoneal inoculation (i.p.) with blood infected via trypomastigotes as a routine procedure in our laboratory. In this study, mice were inoculated i.p. in the left or right lower abdominal quadrant with 200 µL of a solution containing 1000 blood trypomastigotes obtained from another infected mouse, previously quantified in a Neubauer chamber. Fifteen control mice were inoculated with sterile saline solution. Mice were handled according to the ethical considerations in the Mexican Official Standard NOM-062-ZOO-1999 [8].

### 2.3. Parasitemia

Parasitemia curves were determined in the blood collected after the distal cut in the tails. Parasite counts were measured in a Neubauer chamber at 72 h intervals after primary infection, up to day 62.

### 2.4. Histopathological Analysis

After euthanasia, the hearts of 5 inoculated mice and 5 controls, previously perfused with PBS and then with 4% paraformaldehyde (PFA), were removed. A sagittal section of the ventricles was made and preserved in PFA at 4 °C until processing. The samples were dehydrated and paraffin-embedded; then, 4-µm thick sections were cut with a rotary microtome for staining with the hematoxylin-eosin (H.E.), and we used Masson’s trichrome techniques. The prevalence of infiltrate and the presence and number of amastigote nests were determined in all the thickness of the heart in H.E.-stained sections by observing 100 fields under a light microscope with a 40× objective. The presence of uni or multifocal interstitial fibrosis was determined in Masson-stained sections, as well as the extension of collagen fibers, shown by the distinctive blue color.

### 2.5. Classification of Lesions

Based on previous descriptions, initial criteria were defined to determine the presence of thin collagen fibers with a clear background, infiltrate with disruption and/or destruction of adjacent cardiac tissue and/or enlargement of two or more myocardiocytes. Tendon tissue, bundle of His, and atrial and venous endothelia were not considered. The fibrotic lesion found herein was defined as a reactive interstitial fibrosis, characterized by collagen fibers sandwiched between myocardiocytes, with preservation of cardiac structure and function [9,10].

### 2.6. Microphotographs

Microphotographic records were taken with a Canon^®^ camera EOS Rebel T6i (Manufacturer: Canon, Tokyo, Japan) on a Zeiss Primo Star ^®^ microscope (Carl Zeiss AG, Gotinga, Germany) with 10×, 40×, and 100× objectives.

### 2.7. Euthanizing Mice

Mice were sacrificed by CO_2_ exposure. The animals were placed in a clean, empty chamber, and CO_2_ flow was started at a rate of 3 L/min. As gas levels rose to 50%, loss of consciousness was observed by loss of the righting reflex. CO_2_ flow was maintained for at least 1 min after respiratory arrest. Death was verified after euthanasia and prior to disposal, in accordance with official guidelines (NOM-087-SEMARNAT SSA1-2002) [11].

### 2.8. Statistical Analysis

All analyses and charts were performed with Epidat 4.2 (Xunta de Galicia, A Coruña, Spain) and GraphPad^®^ v.7.0 (GraphPad Software, Boston, MA, USA) a for Windows software. The sample size for each group of mice, both experimental and control, was calculated using the software Epidat 4.2, considering an expected difference in proportions between experimental and control animals of approximately 30%, a significance level of 95% and a statistical power of 80%.

The animal study was reviewed and approved by Comisión de Investigación y Ética, División de Investigación, Facultad de Medicina, Universidad Nacional Autónoma de México. Ethic approval code FM/DI/004/2023.

## 3. Results

### 3.1. Infection in a Murine Model

**Figure 1:**
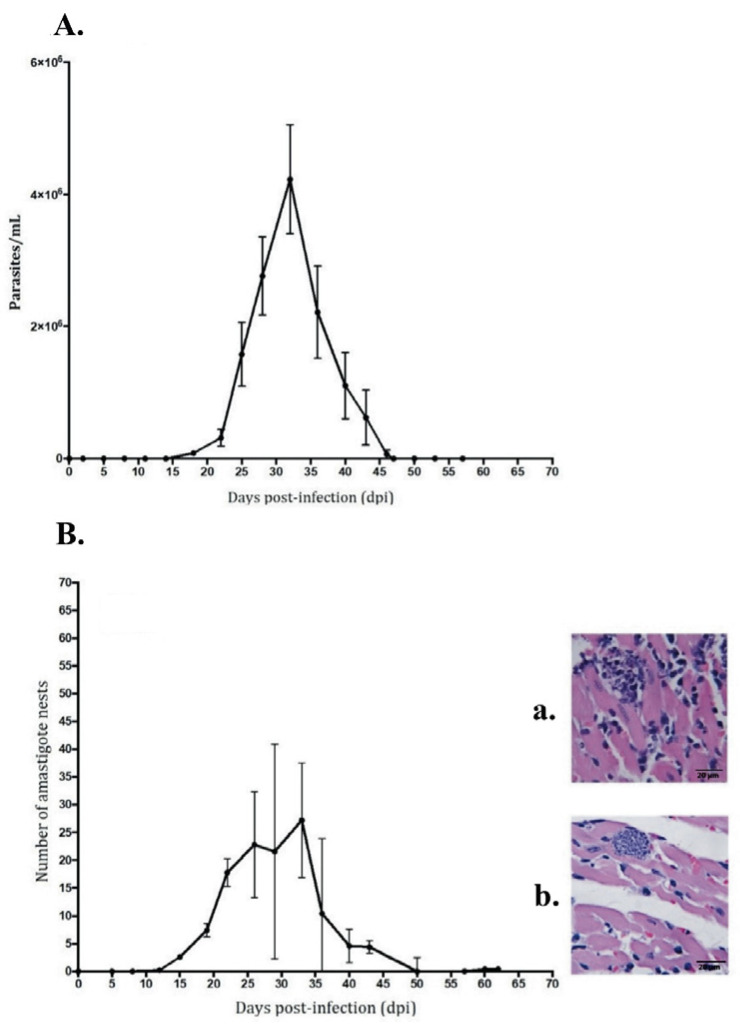
(**A**). Parasitemia, and (**B**). Parasitism. (**A**). Number of parasites. 95% CI of median. Confidence level = 98.08%, 5 replicates of dpi. (**B**). In amastigote nests, CI of median. Two types of nests were found in the myocardium: (**a**) surrounded by infiltrate, and (**b**) without inflammatory infiltrate. 95% CI of median. Actual confidence level, 98.08%. 5 replicates. Inoculum: 1000 parasites. N = 80 mice.

Parasitemia was detectable after 15 dpi, reaching a count of 4.1 × 10^6^ parasites at 33 dpi. According to histopathological analysis, myocardial parasitism began at 15 dpi, with a maximum value of 27 amastigote nests at 35 dpi, with a progressive decrease between 30 and 40 dpi until 62 dpi, when they were no longer detected by the methods used (Figure 1A,B). 

### 3.2. Histopathological Analysis

**Figure 2:**
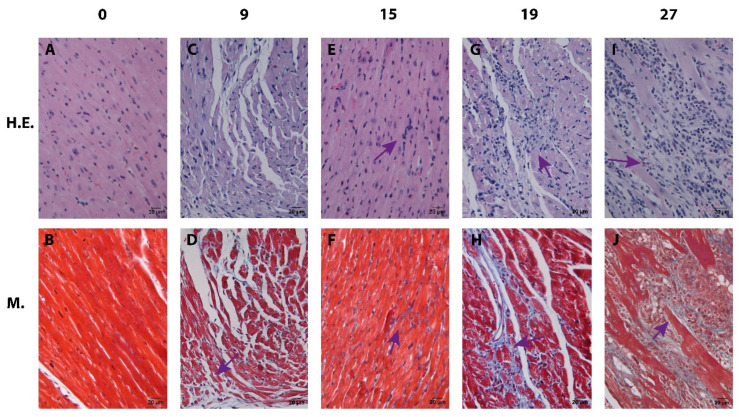
Photomicrographs of cardiac tissue at different times post-infection during experimental murine acute Chagas disease (4000×). H.E.—H.E. stain. M—Masson’s stain. 0 dpi (**A**,**B**)—myocardium-septum: no amastigote nests; normal cardiac histology, no infiltrate, and no lesions. 9 dpi, myocardium-apex: (**C**)—mixed infiltrate (arrow); (**D**)—unifocal lesion with scant infiltrate (arrow). 15 dpi, myocardium-septum: (**E**)—mixed infiltrate (arrow); (**F**)—irregular interstitial fibrosis with scant infiltrate (arrow). 19 dpi, myocardium-right ventricle: (**G**)—mixed interstitial and lymphocytic infiltrate in myocardium and endocardium (arrow); (**H**)—interstitial fibrosis with adjacent infiltrate (arrow). 27 dpi, myocardium-left ventricle: (**I**)—intense inflammatory infiltrate (arrow); (**J**)—multifocal interstitial fibrosis (arrow).

Cardiac tissue at different times (0–27 dpi) where an incipient mixed infiltrate (macrophages, eosinophils and lymphocytes) is observed with the onset of interstitial fibrosis (9–15 dpi). Subsequently, an increase in the cellular and mixed inflammatory response (19–27 dpi) with interstitial fibrotic lesions (27 dpi) (Figure 2).

**Figure 3:**
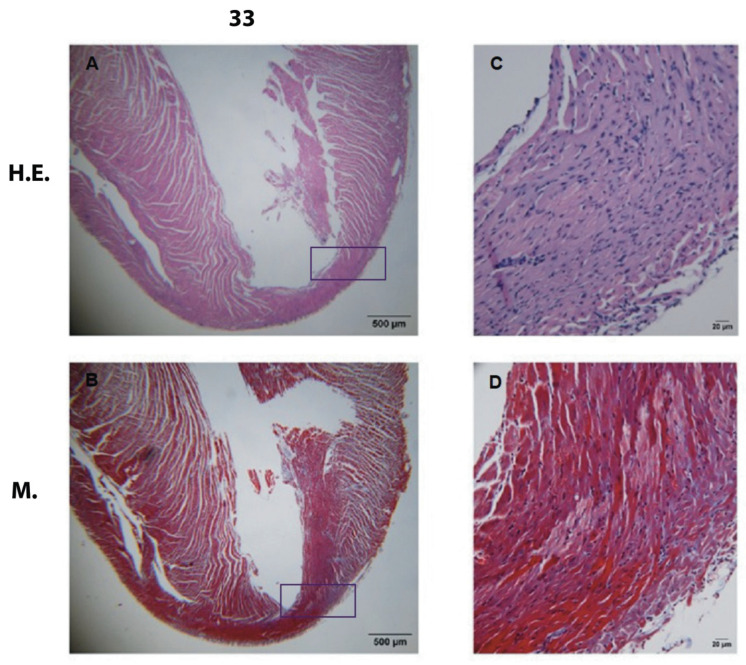
Photomicrographs of heart at 33 dpi. Image at 1000× (left) H.E.—H.E. stain; M—Masson’s stain. (**A**,**B**)—decreased apex is shown, and several areas with interstitial fibrosis (rectangle); (4000× right, rectangle zoom-in) (**C**)— mixed infiltrate, H.E.; (**D**)—presence of extensive interstitial fibrosis.

Organic damage with thinning of the apex (33 dpi) and presence of reactive intestinal fibrosis with mixed inflammatory infiltrate in apex and pericardium (Figure 3).

**Figure 4:**
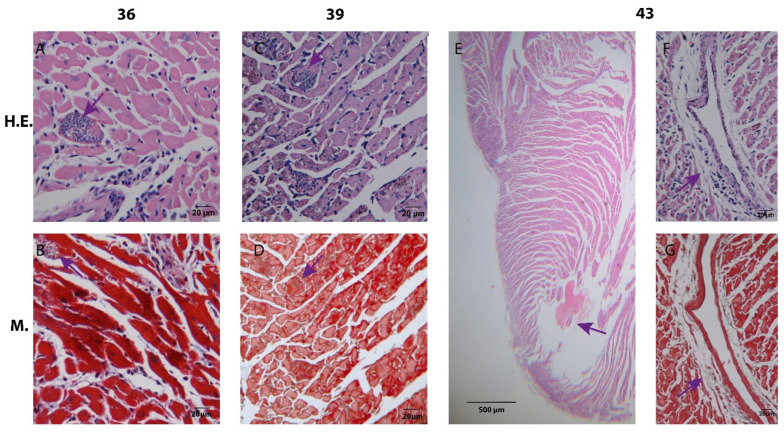
Photomicrographs of myocardium at different times post-infection (4000×) H.E.—H.E. stain. M—Masson’s stain. 36 dpi, myocardium-left ventricle: (**A**)—amastigote nest (arrow). (**B**)—amastigote nest (arrow). 39 dpi, right myocardium-ventricle: (**C**)—amastigote nest and lymphocytic infiltrate (arrows) (1000×). (**D**)—amastigote nest (arrow) (**E**)—43 dpi, intracavitary thrombus proximal to the apex (arrow); 43 dpi (4000×) (**F**,**G**)—perivasculitis, fibrosis, and infiltrate (arrow).

Presence of nests of amastigotes in the myocardium (36–39 dpi). Presence of intracavitary thrombus in the apex (43 dpi) and perivasculitis (Figure 4).

**Figure 5:**
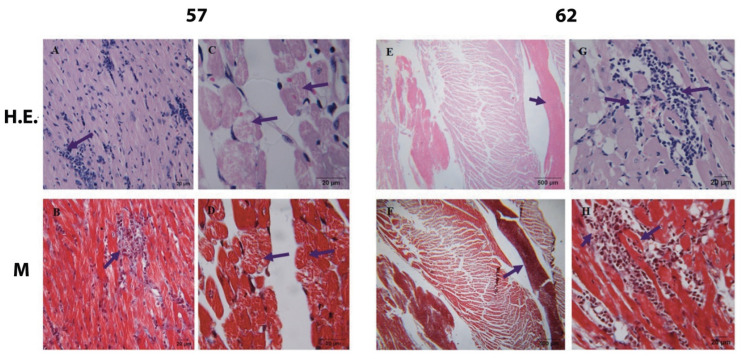
Photomicrographs of mouse myocardium at different times post-infection (4000×). H.E.—H.E. stain. M—Masson’s stain. 57 dpi, myocardium-septum: (**A**)— lymphocyte infiltrate (arrow); (**B**)—lymphocyte infiltrate and interstitial fibrosis (arrow); (**C**,**D**)— vacuolization and myocytolysis (arrows); (**E**,**F**)—62 dpi, endocardium between septum and left ventricle (1000×): intracavitary thrombi in both ventricles (arrow). 62 dpi (4000×), (**G**,**H**)—septum, lymphocytic infiltrate (arrows).

Presence of lymphocytic infiltrate in myocardium with vacuolization and lysis of the myocardium (myocytolysis) (57 dpi). Presence of lymphocytic infiltrate and intracavitary thrombi in both ventricles (62 dpi) (Figure 5).

### 3.3. Composition and Location of Infiltrate

**Figure 6:**
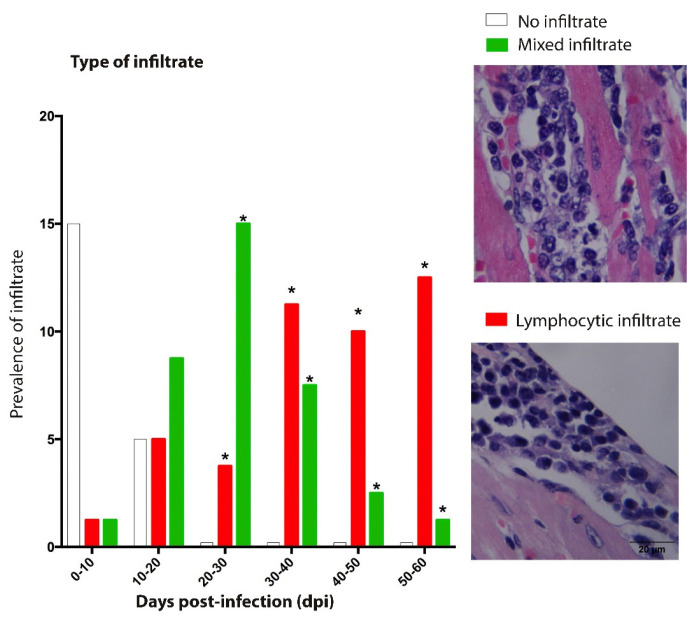
Prevalence of infiltrate in the experimental acute phase of Chagas disease. Mixed-lymphocyte infiltrate with few macrophages and neutrophils. Lymphocytic-only lymphocytes. The increase in lymphocytic infiltrate with respect to mixed infiltrate is noteworthy. * Presence of perivasculitis.

**Figure 7:**
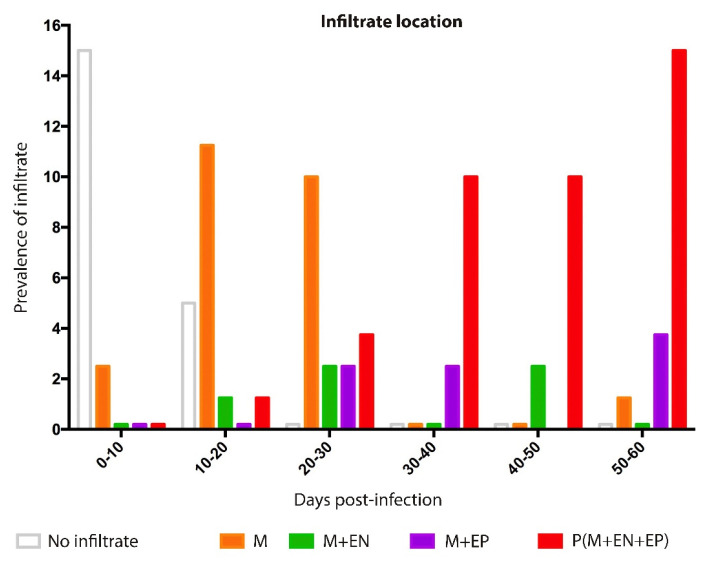
Prevalence and location of infiltrate in heart structures in the experimental acute phase of Chagas disease. M, myocardium; M + EP, myocardium and epicardium; M + EN, myocardium and endocardium; P(M + EN + EP) myocardium, epicardium, and endocardium or pancarditis.

Two patterns of inflammatory infiltrate were identified during the acute phase of infection by *T. cruzi*. First, at 10–30 dpi, the presence of macrophages, eosinophils, and lymphocytes was present mixed infiltrate (Figure 6). Meanwhile, the condition at 30–62 dpi was characterized by the presence of abundant lymphocytes (Figure 5 and Figure 6). The cellular infiltrate was mainly located in the myocardium at 9 dpi, especially at 10–30 dpi; thereafter, the inflammatory process was more frequent in the pericardium (Figure 3 and Figure 6); this resulted in pericarditis (which finally evolved with infiltrate in all three layers of the heart (acute pancarditis) at 50–62 dpi increasing its prevalence (Figure 7); This condition has been described as a cause of death in patients during the acute phase of the disease [10]

### 3.4. Characterizing Fibrotic Damage

**Figure 8:**
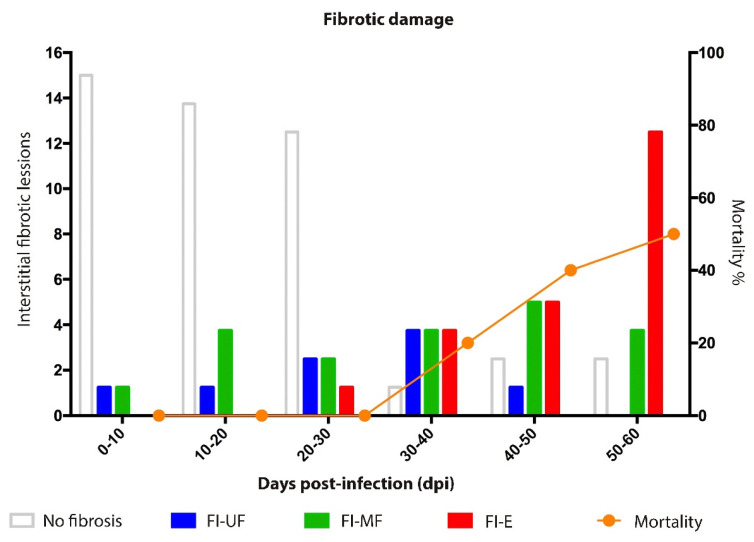
Prevalence of interstitial fibrosis in the experimental acute phase of Chagas disease. FI-UF, unifocal interstitial fibrosis; FI-MF, multifocal interstitial fibrosis; FI-E, extensive interstitial fibrosis. Mortality at 60 dpi; 16-fold increase in FI.5, with 50% mortality in the study model.

**Figure 9:**
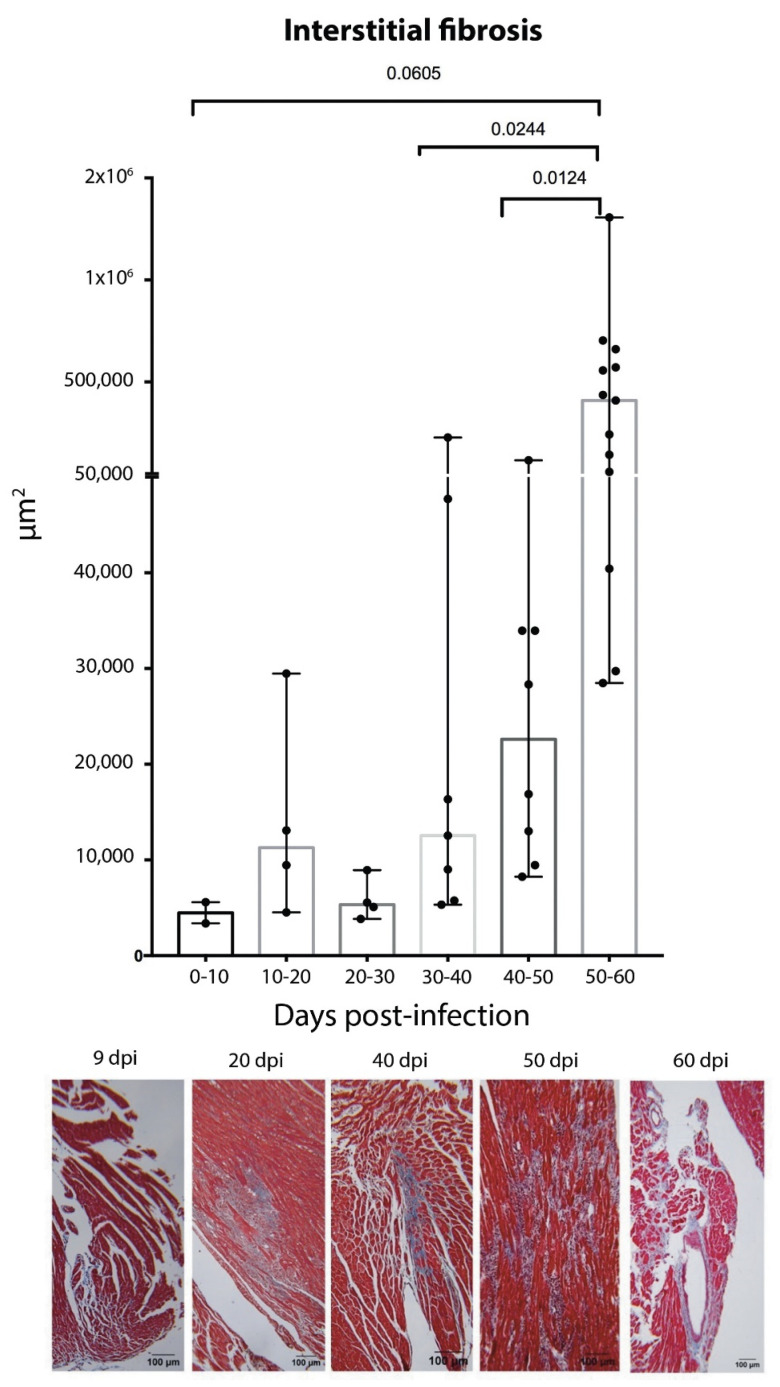
Increase of the areas of fibrotic interstitial lesions at 0–60 dpi in the acute phase. 9 dpi, apex, unifocal lesion. 20 dpi, left ventricle, multifocal lesion. 40 dpi, septum, extensive lesion. 50 dpi, septum, multifocal lesions. 60 dpi, septum, lesions. Differences were analyzed by bilateral unpaired *t*-test. *p* = 0.0033.

Fibrosis is characterized by an excessive production and deposition of collagen fibers to form fibrous connective tissue. Interstitial fibrosis develops between myocardiocytes, with preservation of cardiac structure and function [9,10]. In our study, three types of damage caused by the parasite were observed: (1) Single or unifocal interstitial fibrotic lesion. (2) Multifocal interstitial fibrotic lesion. (3) Extensive fibrotic interstitial lesion (Figure 8). The first observed in a single focus at 9 dpi, this unifocal behavior was found until 19 dpi; then, the prevalence of multifocal lesions increased areas at 30–40 dpi and those of greater extension (Figure 9) became prevalent 50–62 dpi along the mortality in the model (Figure 8).

## 4. Discussion

The acute phase of Chagas disease can vary in duration and clinical presentation. The most common length is 2–3 weeks. WHO indicates a duration of 8 weeks, although it can last up to 4 months [12]; this depends on factors related to patient characteristics, from age to comorbidities, nutritional status, and immune response capacity. The virulence of the strain, the size of the inoculum, and the route of transmission also affect the duration of this clinical phase [12]. 

When the parasite is transmitted by other routes, such as transfusion and even maternal-fetal and to oral transmission, the infection behaves differently from the natural route of entry [13,14]. Acute cases of oral transmission show a greater diversity and severity of symptoms, with high mortality rates due to myocarditis and heart failure [15]. 

The chronic phase lasts 10–20 years. When cardiopathy is diagnosed late and in cases clinically determined to be asymptomatic, without demonstrated pathology, it may even last a lifetime. During the acute phase, nonspecific symptoms begin within 10 days. Affected individuals show signs and symptoms related to the route of entry of the parasite. Parasitemia and amastigote nests in the tissue are evident in this phase. Other nonspecific systemic manifestations such as fever, myalgias, arthralgias or hepato and/or splenomegaly may be present [13,16,17,18,19].

To date, no study has described in detail the histopathological changes that occur during the acute phase of *T. cruzi* infection in a murine model. By considering the genetic variations of the CD1 mouse strain as an experimental model, we observed a behavior similar to that of the disease in humans, where patient populations also show genetic variability [20]. The strain Queretaro, used in this work, was shown to be highly virulent according to Espinoza et al., with mortality rates of up to 100%, this strain shows an evident proinflammatory Th1 response in the murine model [20,21]. Regarding the distribution of *T. cruzi* DTU in Mexico, it has been determined that most strains isolated in Mexico belong to the TcI. Zumaya–Estrada [20,22] described the strain used in this study as part of the North American TcI variant, and Zingales [20,23] noted that TcI strains show preferential tropism toward the myocardium. 

Therefore, in this work, we were able to reproduce the acute phase of Chagas disease in a CD1 mouse model, where the onset of parasitemia was identified at 15 dpi and exponential kinetics was observed at 30 dpi, ending at 45 dpi. Histologically, this phase developed from 22 dpi, and an increase in parasitemia was observed at 40 dpi, ending at 62 dpi (Figure 1A,B). The acute phase in our work lasted 62 days, a time comparable to that described in humans [14].

Therefore, in this work we were able to reproduce the acute phase of Chagas disease in a CD1 mouse model, where the onset of parasitemia was identified at 15 dpi and exponential kinetics was observed at 30 dpi, ending at 45 dpi. Histologically, this phase developed from 22 dpi, and an increase in parasitemia was observed at 40 dpi, ending at 62 dpi (Figure 1A,B). The acute phase in our work lasted 62 days, a time comparable to that described in humans [13].

Regarding the tissue parasitism quantification by PCR and RT-PCR, several authors mentioned some of the limitations that further complicate the choice of primers for quantification of this parasite [24,25,26,27]: high parasitic genetic variability, the considerable size of its genome, which is not completely encoded in the available databases, cross-reactions with other parasites, including *Leishmania* spp. And *Trypanosoma rangeli*, and the geographical specificity of each *T. cruzi* strain. In this regard, Lewis used a fluorescent strain of *T. cruzi* to quantify its presence in vivo and track it directly in cardiac tissue and other organs, who observed results similar to peak parasitism in 30–40 dpi. With an inoculum of 10^3^ parasites, the author also reports that the parasites were microscopically undetectable in blood smears at 35 dpi. As infections progressed, parasite levels were up to 1000 times lower than during the acute stage [28]. The author correlated the bioluminescence in cardiac tissue with approximate values of 10 parasites per 50 ng of DNA at 14 dpi, which at 35 dpi decreased to 10^−1^ in cardiac tissue [28], and finally showed a marked fluorescence in the abdominal region [29]. In a future perspective, a similar study with the Qro. strain could be continued to find the parasitism in other organs.

In toxoplasmosis and amebiasis, among other parasitic diseases, the presence of an infiltrate of monocytes, eosinophils, and NK cells was reported to favor the activation of a Th1-type adaptive immune response [30,31,32]. A typical infiltrate in chagasic myocarditis is mainly composed of macrophages and neutrophils, with little presence of eosinophils and lymphocytes that coexist in early stages [33,34]. In this study, infiltrate foci with macrophages, eosinophils, and neutrophils were identified, which could correspond to the areas where the first binary fissions of the parasite take place [35,36], and which are progressively replaced by a mixed infiltrate (Figure 2, 27 dpi) [36]. These cells are involved in the control of the early infection by phagocytosis. In the murine model used herein, infiltrate foci were identified on the days of highest parasitemia dpi (Figure 1 and Figure 6).

In previous studies, the lymphocytic infiltrate in the chronic phase of Chagas disease was described as being composed of CD4+ and CD8+ T lymphocytes [37]. This response, observed in the acute phase, has a clear proinflammatory profile and is triggered as a host defense, but it has also been linked with the progression of the disease in the chronic phase [34]. Our results show that, at 40–62 dpi, the infiltrate was mainly lymphocytic with few macrophages at 50–60 dpi, the presence of these lymphocytes may be an early signal of the cessation of the acute phase (Figure 6).

Although scarce histopathological reports in cardiac patients indicate a predominance of CD4+ and CD8+ T lymphocytes near the areas of cardiac tissue damage, both lymphocyte types could be involved in tissue pathogenesis [37]. Several authors have reported that CD8+ lymphocytes can cause direct cytotoxicity on myocardial cells by producing granzymes and perforins [38]. As a result of the activation of the cellular response, the iNOS pathway is induced [39] with increased myocardial cytotoxicity. Cabral et al. described that cytokines, such as IFN-γ, are produced by 65–75% of infiltrating cells in the chronic phase, which would maintain an inflammatory response in the absence of amastigotes and without blood parasites [40]; this is consistent with our results, where we observed the presence of lymphocyte infiltrate in the apparent absence of parasitemia and parasitism with the conventional methods.

Notably, we observed the presence of amastigote nests without inflammatory cellular infiltrate (Figure 4, 36 dpi). Several authors reported amastigote nests without cellular infiltrate and suggested deficiency in signaling for leukocyte recruitment, a condition that allows *T. cruzi* to evade the immune response. It has been suggested that these nests may be latent inside the cell, as described by Texeira in 2011, as “hypnomastigotes” within non-phagocytic muscle cells, where they can persist for decades [41,42,43].

In this study, a progression was observed in the severity and location of cellular infiltrate. It was first found in the myocardium, then in the myocardium and endocardium and, finally, encompassing all three heart layers. Clinically, this is described as acute pancarditis and was reported as a cause of sudden death in the acute phase of Chagas disease. Some authors have reported no clinical manifestations in these deceased patients; however, extensive lymphocytic infiltrates have been described in autopsies of human cases and dogs [43,44,45]. In our study, infiltrates were identified in the epicardium, which suggests that severe cases of acute pericarditis may occur from the onset of infection (Figure 3 and Figure 7) [46].

While there are few reports of histopathological alterations, the presence of extensive lymphocytic infiltrates, as well as mononuclear infiltration and lysis of muscle fibers, has been documented in *post mortem* examinations [45]. Similar findings were also reported in autopsies of dogs diagnosed with severe acute myocarditis [44].

Along with infiltration of immune cells, the deposition of collagen fibers on the endothelial layer of blood vessels was observed, causing a remarkable perivasculitis at 29–62 dpi (Figure 4, 43 dpi, arrows). Perivascular fibrosis also plays a role in cardiac hypertension, as it leads to impaired blood flow, hindering the supply of oxygen and nutrients to cardiac tissue [46]. Moreover, these lesions are usually described in the chronic phase of the disease. Thus, inflammatory infiltrate and endothelial cells is involved in perivascular inflammation, which may result in vasospasm and microvascular thrombosis in the endothelium, and to the cardiac damage observed in the acute phase.

A decrease in the number of amastigote nests was observed at 57–62 dpi, along with undetectable parasitemia by the methods used; a marked vacuolization of myocytes, the so-called myocytolysis [47,48,49], was observed in this period (Figure 5, 62 dpi, arrows). This condition coincides in time with the development of lesions in the micro- and macrocirculation, which leads to ischemia and may cause myocytolysis [50]. Other authors have described myofibrillar degeneration in this phase, with disappearance of Z bands in some myocardial fibers and cellular atrophy, which could correspond to the myocytolysis herein described [51,52].

### 4.1. Macroscopical Findings

A greater number of amastigote nests were observed in the tissue at 30–40 dpi. Macroscopically, the heart showed frank dilation due to hypertrophy (Figure 3A and Figure 4E). These changes were related to the presence of mixed inflammatory and lymphocytic infiltrate in the three layers of the organ (Figure 6) and the presence of incipient interstitial fibrosis (Figure 8 and Figure 9). This favored the enlargement of the interstitial space, where myocardial fibers probably began to develop muscle atrophy [53]. These processes increase intracavitary ventricular pressure during systole, which results in a decrease in cardiac output. Echocardiographic studies show a decrease in left ventricular ejection fraction [54]. This alteration in hemostasis can lead to the formation of intracavitary blood clots [55], which were observed herein after 43 dpi (Figure 4E and Figure 5).

### 4.2. Fibrosis 

The presence of collagen fibers and cellular infiltrate is observed in early stages of infection; our results suggest that tissue damage caused by the parasite and tissue remodeling processes occur simultaneously. The histopathological studies in this work confirm the progression of interstitial fibrosis, which evolves into extensive lesions (Figure 9); these are probably precursors of the cicatricial fibrosis often described in the chronic phase of Chagas disease [56]. Herein, the fibrosis reported may depend on the presence of the parasite, the inflammatory process, vascular damage, thrombus formation, and the presence of myocytolysis in the cardiomyocytes, which jointly lead to fibrosis and also determine the survival of the model (Figure 8). On this regard, the presence of this incipient extensive interstitial fibrosis was observed in our murine model, where lesions in the acute phase could be manifested in asymptomatic human cases as incipient lesions, detected as decreased parietal motility in ECO studies, which also show parietal hypertrophy as an expression of the cellular inflammatory process [57], which, in these cases, showed a proinflammatory cytokine profile [58].

## 5. Conclusions

This work on a CD1 murine model allowed, for the first time, a successive study of the histopathological processes associated with the acute phase of infection by *T. cruzi*, which may contribute to a better understanding of this phase in humans.

The presence of the parasite compromises cardiac function early as a result of the interaction between the parasite and the immune system, which triggers various histopathological alterations during the acute phase.

During the acute phase, the lymphocyte infiltrate found at early times in the myocardium could be involved in both the defense and the maintenance of the inflammatory response associated with the pancarditis observed at the end of this study.

**Figure 10:**
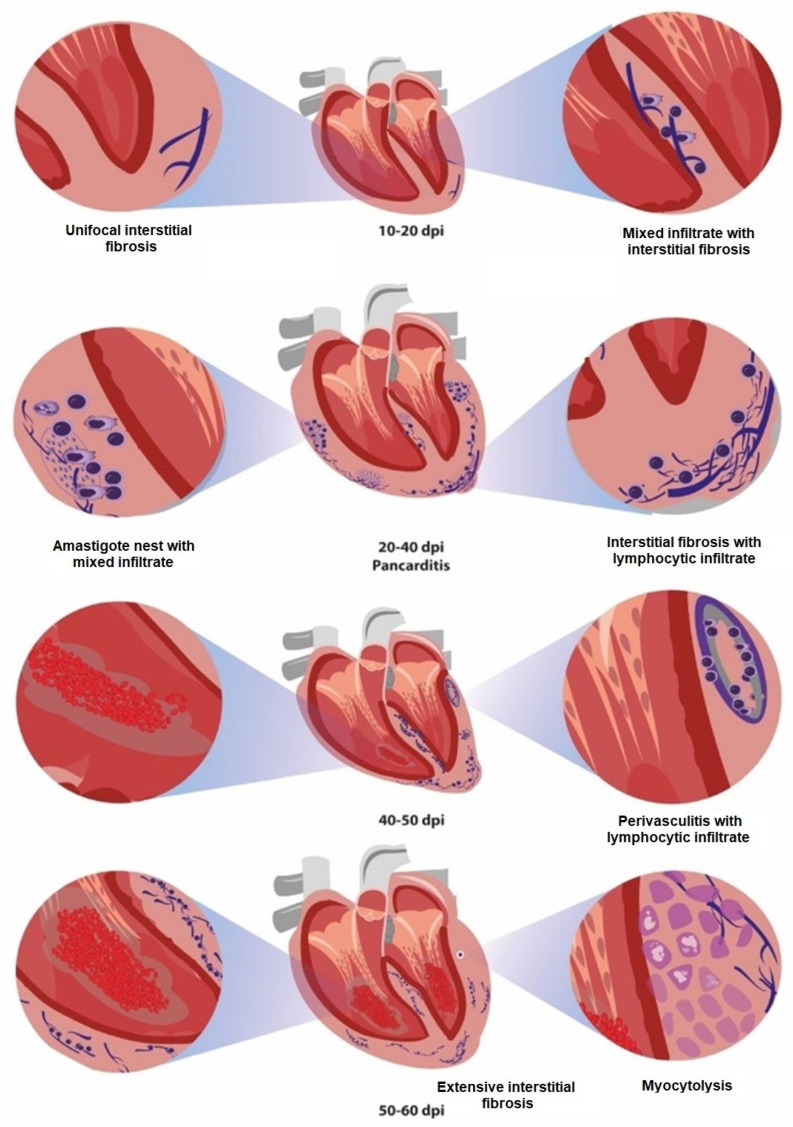
Model for the presence of histopathological alterations in acute murine experimental *T. cruzi* infection. Summary of the progression of histopathological lesions observed. dpi, days post-infection.

Our results indicate, for the first time, the presence of cardiac interstitial fibrosis in the acute phase. Cardiac fibrosis was described as being characteristic of chronicity in Chagas disease; however, in our study, it was initially identified as unifocal interstitial fibrosis at 9 dpi, which progressed to the extensive interstitial fibrosis typical of the chronic phase (Figure 10).

Thrombosis has not been reported as a complication of the disease during the acute phase of the infection. This highlights the importance of an early cardiological diagnosis, which would require the inclusion of therapeutic approaches for these cases and to prevent concomitant thrombotic and thromboembolic events in infected patients, which are usually not considered in this phase.

Perivasculitis and pancarditis are poorly studied in the pathogenesis of lesions in the acute phase of Chagas disease.

The presence of amastigote nests, without cellular infiltration, suggests an inhibition of the immune response or encryption promoted by *T. cruzi*: a mechanism that should be further studied.

## Data Availability

Not applicable.
